# Metastable Nanobubbles

**DOI:** 10.1021/acsomega.0c05384

**Published:** 2021-03-16

**Authors:** Tapio Vehmas, Lasse Makkonen

**Affiliations:** VTT Technical Research Centre of Finland, P.O. Box 1000, 02044 VTT Espoo, Finland

## Abstract

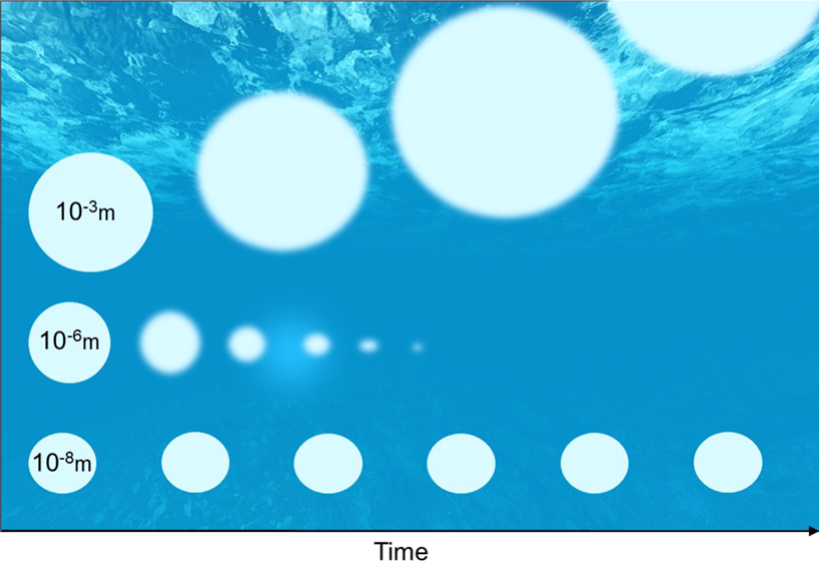

Water containing suspended nanobubbles is utilized in various applications. The observed lifetime of suspended nanobubbles is several weeks, whereas, according to the classical theory of bubble stability, a nanosized bubble should dissolve within microseconds. Explanations for the longevity of nanosized bubbles have been proposed but none of them has gained general acceptance. In this study, we derive an explanation for the existence of metastable nanobubbles solely from the thermodynamic principles. According to our analysis, the dissolution of nanosized aqueous bulk bubbles is nonspontaneous below 180 nm diameter due to the energy requirement of gas dissolution. Hydrophobic surfaces have a further stabilizing effect, and the dissolution becomes nonspontaneous in surface nanobubbles having a diameter below 600 nm.

## Introduction

The lifetime of nanosized bubbles has been in focus of active debate since 1990s. In 1994, Parker et al. published observations of nanobubbles on hydrophobic surfaces.^1^ According to the conventional theory, bubbles having a size in the nanometer scale should dissolve rapidly.^2^ Following the initial observation, nanobubbles were observed on hydrophobic surfaces in multiple studies.^3−6^ Today, the existence of nanobubbles on hydrophobic surfaces is inarguable.^7,8^

The existence of nanobubbles is not restricted to hydrophobic surfaces, as several studies have reported nanosized bubbles in bulk solution. In 2010, Ohgaki et al. published results of nitrogen, methane, and argon nanosized bubbles in bulk solution.^9^ They found that the lifetime of nanobubbles was over 2 weeks and the average radius of the bubbles was 50 nm. Later, Ushikubo et al. also reported nanosized oxygen, xenon, and air bubbles in a bulk solution based on measuring the size distribution with dynamic light scattering and confirming the gaseous nature of the bubbles with nuclear magnetic resonance.^10^ More recently, Jin et al. studied nanobubble evolution with dark-field microscopy and concluded that nanobubbles can be formed from microbubble collapse.^11^ Nanobubbles were also studied with respect to applications and biological processes.^12,13^

The experimental evidence of nanosized bubbles has generated various theories of nanobubble stability. These theories have either focused on explaining the stability of nanosized bubbles on hydrophobic surfaces^14^ or providing a more universal explanation of bubble stability, also accounting nanobubbles in bulk solution.^15^ Brenner and Lohse proposed a dynamic equilibrium model to explain the longevity of surface nanobubbles.^16^ In their model, the diffusive gas outflux is exactly balanced by the gas influx. Ducker explained the surface bubble stability by contaminants that limit the diffusion at the bubble boundary.^17^ Weijs et al. proposed that nanobubble longevity originates from diffusive shielding.^18^ Recently, Manning proposed that the discontinuous pressure change over the gas–liquid interface, predicted by the Young–Laplace equation, prevents the formation of a concentration gradient in the liquid.^19^

Matsumoto and Tanaka^20^ studied stability conditions of bulk nanobubbles and concluded that, under vacuum or high tensile stress, bulk nanobubbles are stable.^20^ Ohgaki explained nanobubble stability with hard hydrogen bonds in the bubble surface.^9^ Zhang stated that nanobubble longevity originated for high internal density.^21^ In 2020, Tan et al. proposed ζ-potential as an explanation of bulk nanobubble longevity.^22^ Michailidi et al. attributed nanobubble stability to hydrogen-bonding interactions on the bubble surface.^23^

None of these theories has gained general acceptance. The most widely accepted theory for surface nanobubbles explains bubble stability on hydrophobic surfaces by a pinning effect.^24,25^ According to this theory, pinning of the triple point of gas, substrate, and solution, by surface impurities and/or defects, causes additional force that hinders the bubble dissolution. Although pinning is a fundamental aspect of triple-point dynamics^26^ and this theory has gained some acceptance, it fails to provide an explanation to nanosized bubble stability in bulk solution. It also remains unexplained how triple-point pinning can prevent the previously assumed dissolution of pressurized gas at the gas–liquid interface.

Classical theories do not predict long lifetimes of nanosized bubbles. According to the well-known Young–Laplace equation (eq 1), the pressure inside the bubble is higher than in the liquid due to surface tension (σ). The generally accepted view is that this high pressure causes a rapid dissolution of nanobubbles. This theoretical paradigm is often stated as: “the air inside the nanobubble cannot be in equilibrium with the surroundings since larger pressure means larger chemical potential, and consequently, the nanobubble should dissolve”.^27^ However, this paradigm involves a conceptual discrepancy. If the high pressure (*p*) inside a nanobubble is the cause of the dissolution, then logically, *p* should decrease upon dissolution. However, eq 1 shows that when a dissolving bubble decreases in size, *p* increases. It is, therefore, obvious that the stability analysis must be based on a more comprehensive understanding of bubble thermodynamics.
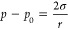
1Bubble lifetime and stability have been theoretically analyzed in the past decades. In 1950, Epstein and Plesset calculated bubble lifetimes related to the diffusion of dissolved gas from a bubble boundary.^28^ According to these calculations, a nanosized bubble should dissolve within microseconds. However, a large bubble tends to grow due to the Kelvin effect. The Kelvin effect describes the vapor pressure over a curved surface (eq 2). Vapor pressure of a large bubble has a significant contribution to the bubble pressure, causing large bubbles to grow.
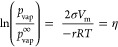
2Chemical potentials of bubbles and the surrounding solution must be considered to introduce the Kelvin equation to the bubble dissolution model. The derivation of bubble stability from chemical potentials was performed by Ward et al.^29^ According to their model, larger bubbles grow in size and smaller bubbles dissolve. The limiting radius is obtained from eq 3, and at atmospheric pressure and saturated conditions, it is 250 μm for an aqueous bubble. Equation 3 can also be deducted from the Epstein–Plesset theory by introducing the vapor pressure term. The theory has been experimentally verified, albeit only for bubbles larger than 5 μm.^30^
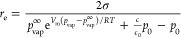
3

## Results and Discussion

As discussed above, in the classical thermodynamic and chemical potential view, nanosized bubbles should not exist. However, a high chemical potential does not necessarily mean that the process will happen spontaneously. For a process to take place spontaneously, the process must be instantaneously energetically favorable. Gibbs energy is a useful tool to evaluate if a process happens spontaneously. If Gibbs energy decreases freely in a process, the process is spontaneous. If Gibbs energy change is positive, external energy is needed to facilitate the process. The external energy that is required to initiate a process is called activation energy and is a well-known concept in chemistry.^31^

In the current approach, ideal behavior of the gas and constant temperature are assumed. In ideal gas, there are no interactions between the gas molecules so that the internal energy of the gas is independent of the molecule separation and, hence, independent of the volume of the sample (eq 4). This assumption leads to the definition of perfect gas (eq 5). Gas dissolution according to Henry’s law is also assumed. Henry’s law states that dissolved gas concentration is linearly proportional to the gas pressure and vice versa (eq 6).
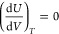
4
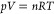
5
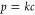
6The studied system consists of a gas bubble, surrounded by liquid bath. The total volume of the system is *V*_total_, which consists of the volume of the gas bubble and the volume of the surrounding solution (eq 7). Pressure in the solution phase is constant (*p*_0_) and the pressure of the gas bubble is the sum of internal gas pressure and vapor pressure (eq 8).
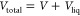
7
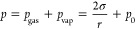
8The system consists of an internal gaseous phase of the bubble, dissolved gas in the solution, gas/solution interface, bulk liquid, and vapor inside the bubble. In the following, the Gibbs energy of each of these phases is derived separately. The total Gibbs energy of the system is the summation of the Gibbs energy of each phase (eq 9).
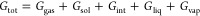
9When a decrease in the bubble radius causes a negative change in the total Gibbs energy, the bubble dissolves spontaneously (eq 10).
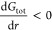
10Bubble dissolution inflicts changes in the phases outlined above. In the internal gaseous phase, bubble dissolution reduces the bubble volume, and due to surface tension, the pressure of the remaining gas increases. Gibbs energy change related to the gaseous phase inside the bubble is
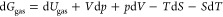
11As ideal gas behavior and constant temperature in the system are assumed, the Gibbs energy term reduces to the terms related to changes in volume, internal pressure, and entropy change
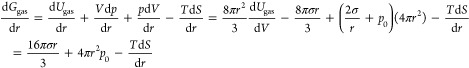
12Over a flat gas/liquid surface, the pressure in the gaseous and solution phases is the same. As the gas solubility is proportional to the pressure, the dissolved gas phase in the solution is never supersatured over a flat surface during gas dissolution. Over a curved surface, however, there is a pressure difference between gaseous and solution phases (eq 8). In the case of a bubble, the pressure difference between gaseous and solution phases leads to supersaturation of dissolved gas during the bubble dissolution. This supersaturation creates the concentration gradient in the liquid that is necessary for transferring dissolved gas from the interface to the liquid. Although the ambient pressure is lower than the pressure inside the bubble, the generation of supersaturation requires energy. This energy has not been taken into account in the previous attempts to explain the stability of nanobubbles.^32^

**Figure 1:**
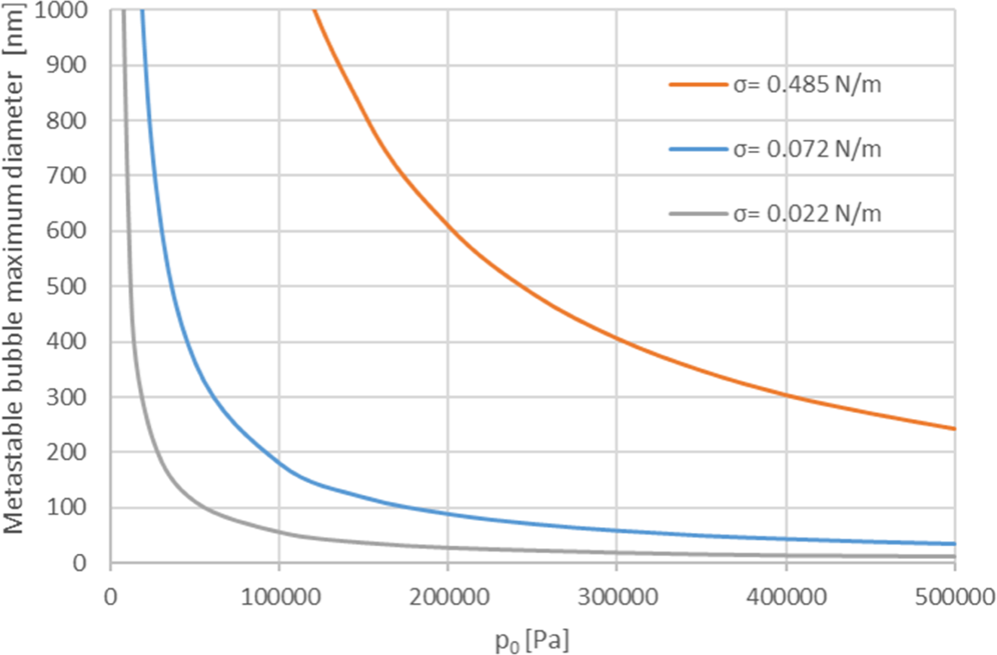
Maximum diameter of a metastable nanosized bubble in ethanol (σ = 0.022 N/m), water (σ = 0.072 N/m), and mercury (σ = 0.485 N/m) as a function of the external pressure.

The dissolution of a bubble increases supersaturation of the surrounding solution. The Gibbs energy related to the dissolution of the gaseous phase into the surrounding solution is
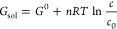
13and its change is
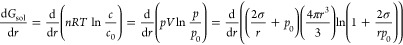
14Bubble dissolution reduces the surface area and hence the surface energy of the bubble. The Gibbs energy change related to surface area is
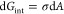
15and the Gibbs energy change related to a change in the bubble radius is
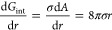
16The Gibbs energy term related to bulk liquid is
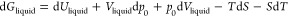
17As the external pressure is assumed constant and the volume of the bulk liquid is independent of the bubble radius, only entropy change is related to bubble radius at a constant temperature
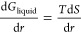
18The Gibbs energy change related to the vapor pressure inside the bubble is
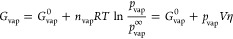
19The Gibbs energy change of vapor pressure related to bubble dissolution is
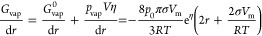
20The total change in the Gibbs energy is obtained by combining the terms above with eqs 9 and 10, which leads to eqs 21 and 22.
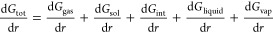
21
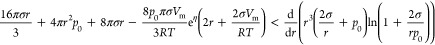
22A graphical presentation of eq 22 is shown in Figure 1 for three solid–liquid surface tension values, corresponding to water, ethanol, and mercury.

According to eq 22, a bubble having a diameter smaller than 180 nm does not dissolve spontaneously in water at normal temperature and pressure. Such bubbles are not in thermodynamic nor chemical equilibrium and they are energetically at a higher level than the surrounding environment. Nevertheless, bubble dissolution does not take place spontaneously because activation energy is required to initiate the process. This observation removes the nanobubble paradox. A high bubble pressure and a higher chemical potential do not inflict the dissolution of gas from the bubble. The classical intuitive paradigm of the root cause of bubble dissolution is not correct, according to the derivation presented above.

**Figure 2:**
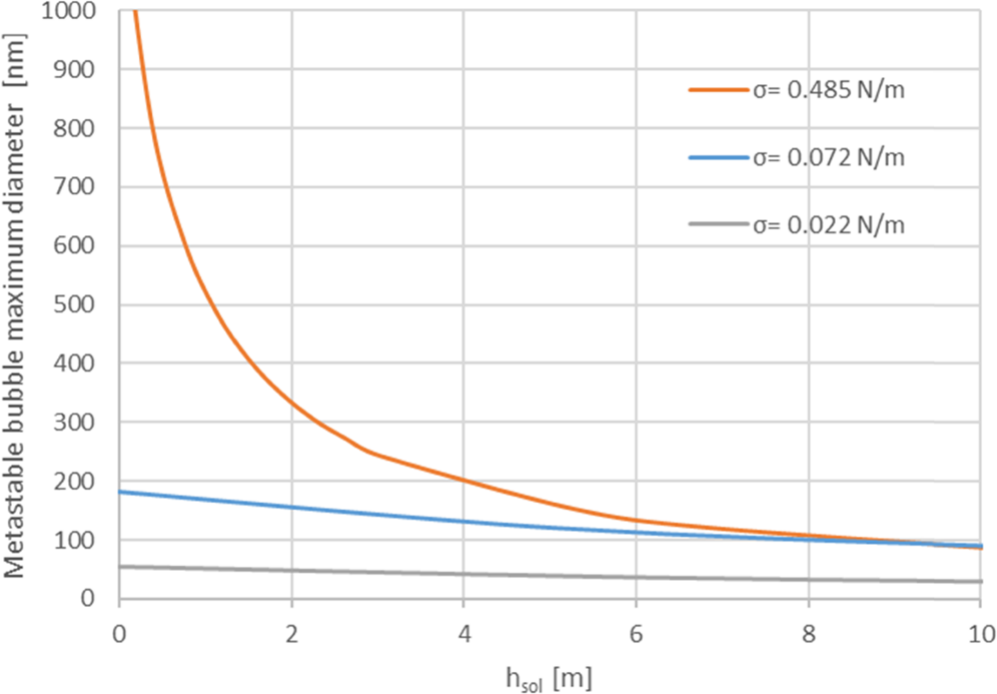
Maximum diameter of metastable nanosized bubbles in ethanol (σ = 0.022 N/m), water (σ = 0.072 N/m), and mercury (σ = 0.485 N/m) as a function of solution depth (*h*_sol_).

Adding hydrostatic pressure to the external pressure (eq 23) enables estimation of bubble stability as a function of the solution depth.
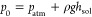
23Figure 2 shows the maximum diameter of metastable bubbles as a function of solution depth, accounting for the hydrostatic pressure of the solution. According to these results, at high depths in natural water bodies, only very small nanobubbles can exist.

**Figure 3:**
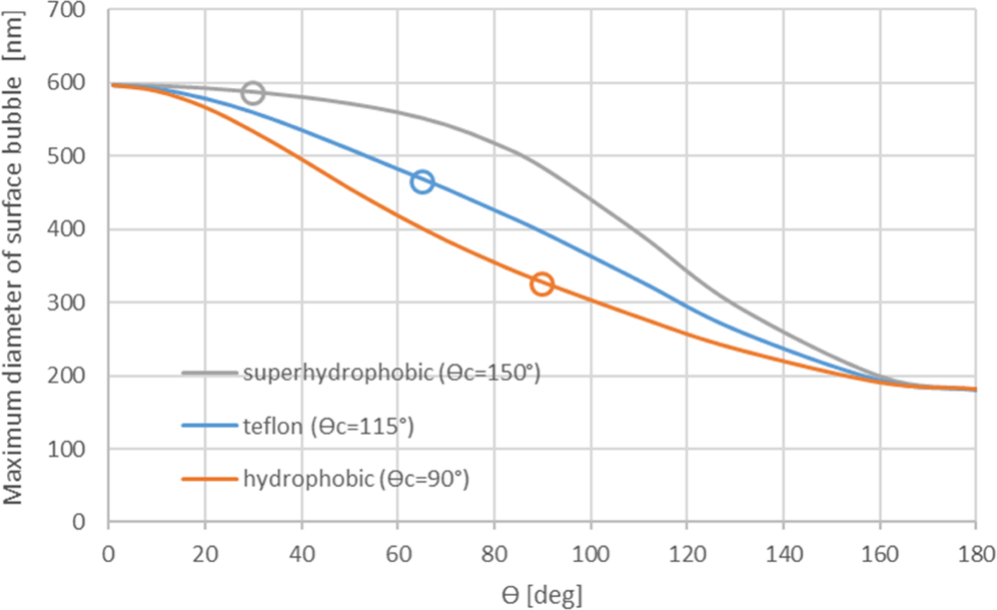
Maximum diameter of metastable nanosized surface bubbles on a hydrophobic surface (θ_c_ = 90°), Teflon surface (θ_c_ = 115°), and superhydrophobic surface (θ_c_ = 150°) as a function of the bubble contact angle (θ). Circles represent the equilibrium shape of a bubble on the surface.

The theory presented above is also applicable to surface bubbles when the effect of solid/gas and solid/liquid surface energies is included in the equation. Surface interaction with gas and solution is characterized with contact angles. Surface energies of gas/solid, gas/liquid, and solid/liquid interfaces obey Young’s equation.
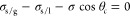
24The surface area of the gas/solid interface under the bubble is related to the radius of the curvature of the bubble and the contact angle of the bubble according to eq 25. The volume of the surface bubble and area of the gas/liquid interface are shown in eqs 26 and 27, respectively.
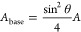
25
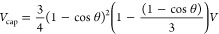
26
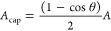
27On a smooth surface, a bubble is attached to the surface at the gas/solid interface. The surface energy change related to the formation of the gas/solid interface is
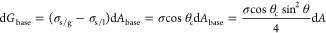
28
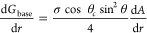
29The energy terms related to the internal gaseous phase, gas/solution interface, and the dissolution of the internal gaseous phase are derived in eqs 30–32, respectively.
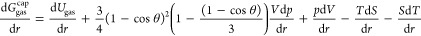
30
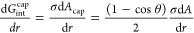
31
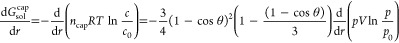
32The limiting condition of metastability is
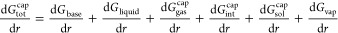
33The equation for the size limit of metastable surface bubbles is obtained by inserting eqs 18, 20, 29, 30, 31, and 32 into eq 33, and solving numerically. The size-calculated limits of metastable nanobubbles on three different smooth surfaces in an aqueous solution are shown in Figure 3.

In Figure 3, the equilibrium bubble contact angles are shown as circles. According to Figure 3, surface bubbles having a lower contact angle than the equilibrium angle are more stable. This observation is in qualitative agreement with experimental surface bubble studies, where lower contact angles are often measured. The pinning force that prevents a bubble from returning to its equilibrium angle is not the reason for the bubble stability, according to the derivation presented above.

### Comparisons with Experimental Data

According to the theory derived above, nanosized bubbles having a diameter below 180 nm are metastable in water at room temperature. Aqueous nanobubbles have been produced in bulk liquids with multiple methods, and the sizes of the bubbles were analyzed. Ma et al.^33^ used a porous alumina film to generate nanobubbles and measured bubble sizes using nanoparticle tracking analysis (NTA). NTA utilizes Brownian motion of ultrafine objectives to determine the size of the particle. NTA is considered a more accurate method to determine the true ultrafine objective size than dynamic light scattering, which measures the hydrodynamic radius of the particle. Ma et al. present bubble sizes up to the lifetime of 9 days. According to this study, bubble size increased due to coalescence of the bubbles during the observation period. Most of the measured sizes were below the theoretical 180 nm diameter. On the 9th day of the measurements, the particle sizes showed a large abruption around 180 nm. Some ambiguous particles that are too large to support the theory were then observed. However, as the amount of these particles does not seem to fluctuate as that of the bubbles, they may have been solid particles.

Azevedo studied aqueous nanobubbles in α-terpineol solutions and measured object sizes with NTA.^34^ In distilled water, diameters of the bubble were around 180 nm, corresponding to the theory. In an α-terpineol solution, having a surface tension of 49 mN/m, measured bubble diameters were 120 nm. The maximum size of the bubbles with a surface tension of 49 mN/m is 123 nm according to the proposed theory, which is in agreement with the measurements.

Ke et al. used a compression–decompression method to produce nanobubbles and studied bubble sizes using NTA.^35^ Most of the measured bubbles were around 180 nm in diameter, some pressure-treated bubbles having a smaller diameter (140 nm). Some larger diameters were observed but they were not abundant in the solution, indicating impurities. Tuziuti et al. studied the effect of pressure changes in commercially available nanobubble water^36^ and measured particle sizes with laser diffraction/scattering. Initial bubble size was 110 nm and, after the pressure treatment, around 180 nm, in agreement with our theoretically derived maximum bubble size. Thus, data that support the maximum theoretical metastable radius of around 180 nm seem to be abundant.^37,38^ However, measurements related to the hydrodynamic radius are above the maximum theoretical value of the theory,^10,39,40^ which may be due to inaccuracy of such measurements.

As to surface nanobubbles, this theory proposes that bubbles on hydrophobic surfaces with a diameter below 600 nm are metastable at atmospheric pressure. However, the literature suggests that surface bubbles up to multiple micrometer-scale “pancakes” are also sometimes observed.

Zhang et al. measured contact angles and the height of the surface bubbles on mica and highly oriented pyrolytic graphite (HOPG) surfaces, using air and hydrogen.^41^ Contact angles of the air bubbles on mica surfaces ranged from 25 to 60°, having heights 20 to 65 nm, respectively. According to the theory proposed here, surface bubbles having a contact angle of 25° on a mica surface are stable up to 25 nm height, and bubbles having a contact angle of 60° are stable up to 90 nm height. The maximum height measured by Zhang et al. was close to 90 nm. However, their reported contact angle of 40–45° is not in the range of stable surface bubbles according to the theory. On an HOPG surface, our theory proposes stable bubbles with contact angles of 10 and 30°, having a maximum height 4 and 37 nm. The measured heights were 10 and 20 nm, which are close to the predictions of the theory, but another observed data group, having height up to 60 nm, is not stable according to the theory. In another study, Zhang et al. measured contact angles and heights of the surface nanobubbles in a 0.5 CMC Tween solution (surface tension 40 mN/m).^42^ Their results are close to the calculated theoretical values with a lower set point amplitude ratios (contact angle 33°, measured height 17 nm, and calculated maximum 24 nm). The proposed theory also predicts the maximum bubble sizes in formamide, ethylammonium nitrate, and propylammonium nitrate quite well.^43^ The comparisons described above are summarized in Table 1.

**Table 1:** Comparison of Experimental and Calculated Surface Bubble Sizes in Various Solutions and on Different Surfaces

observed bubble size [nm]	maximum theoretical stable bubble size [nm]	solution	gas	surface	ref
60, 80, 110, 140	180	aqueous	CO_2_, N_2_, O_2_, He, Ar		(31)
180	180	aqueous	air		(32)
120	123	α-terpineol	air		(32)
140, 180	180	aqueous	N_2_, Kr		(33)
110, 180	180	aqueous	air		(34)
20/65	25/90	aqueous	air	mica	(41)
10/20	4/37	aqueous	air	HOPG	(39)
17/-	24/-	CMC Tween solution	air	OTS silicon	(40)
15/70	78/232	formamide	air	HOPG	(41)
15/70	64/202	ethylammonium nitrate	air	HOPG	(41)
10/75	55/166	propylammonium nitrate	air	HOPG	(41)

## Conclusions

**Figure 4:**
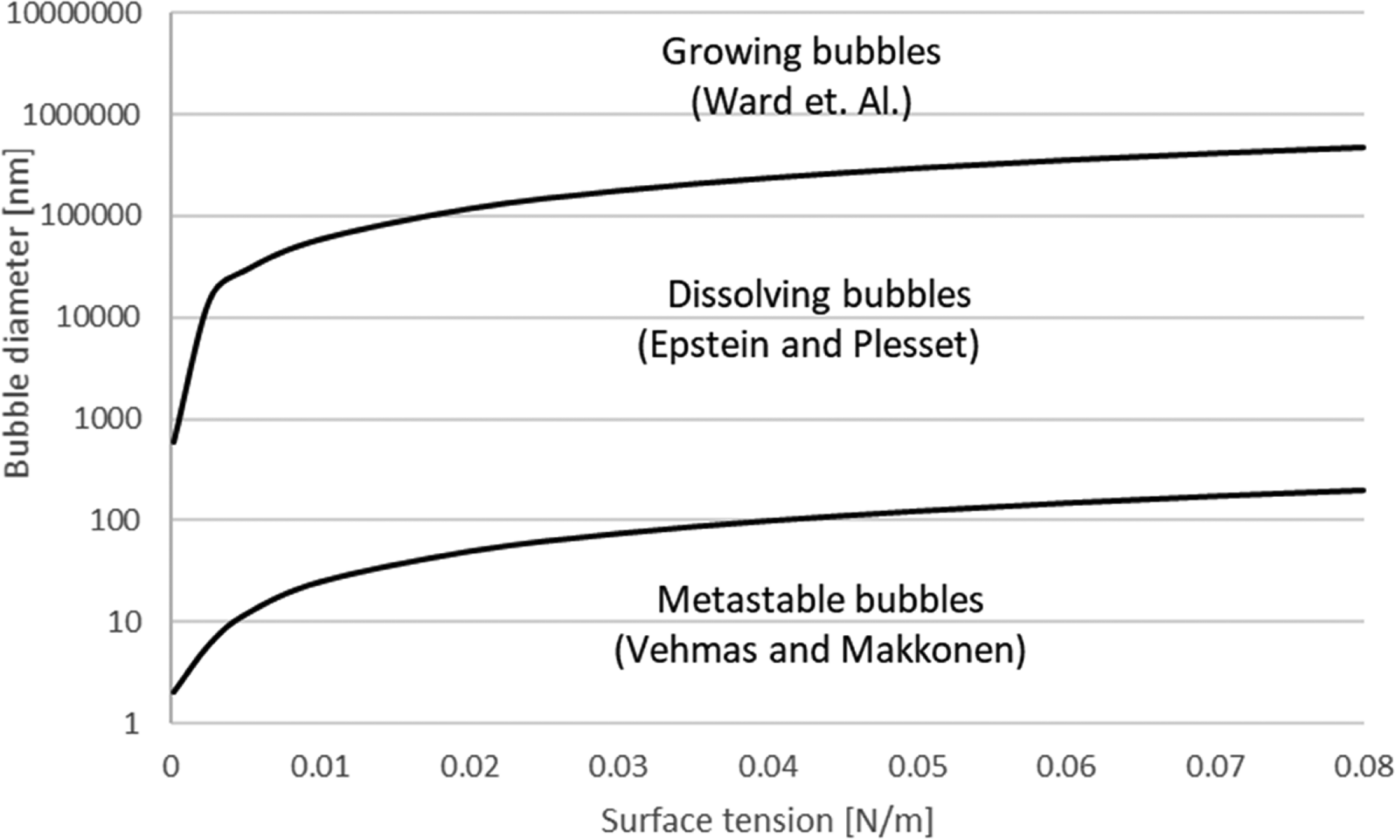
Behavior of bubbles as a function of solution surface tension and corresponding theories.

We showed above that metastability of a nanosized bubble can be derived from the fundamental equations of thermodynamics, without adding external quantities, forces, or anomalies. This shows that the widely spread intuition that the high pressure inside a nanobubble causes its quick dissolution is incorrect. Such a process is not initiated spontaneously because the dissolution of a bubble does not decrease the pressure inside it. A proper solution to the nanobubble stability requires a rigorous treatment of all terms of the total Gibbs energy change. An illustration of bubble behavior in saturated solutions with various surface tensions is presented in Figure 4.

According to the thermodynamic analysis presented in this paper, at room temperature and pressure, an aqueous bulk bubble having a diameter below 180 nm does not begin to dissolve spontaneously. In ethanol, the corresponding limit is 55 nm, and in mercury, 1220 nm. Decreasing the external pressure increases the limit of a metastable bubble diameter.

The theory presented in this paper is also applicable to surface bubbles. According to the theory, surface nanobubbles can be stable in saturated solutions, in contrast to earlier analysis.^32^ The hydrophobicity of a surface increases the maximum diameter for a stable surface nanobubble. In water, the maximum diameter of a stable surface bubble varies between 180 and 600 nm depending on hydrophobicity. An aqueous bubble on a Teflon surface has an equilibrium form having a diameter of 480 nm and the contact angle of 60°. Bubbles with contact angles below the equilibrium contact angle are more stable, which is in qualitative agreement with the observed nanosized bubble behavior on hydrophobic surfaces.

## Experimental Section

Experiments were also made in-house to verify the presented theory for bulk nanobubbles. A commercially available micro-/nanobubble generator (Asupu BA06S) was used to prepare air nanobubbles in ion-exchanged water. According to manufacturer’s specifications, the mean diameter of the generated bubbles is 55 nm.^44^ The generator consists of a ceramic lamellae pump (Fluid-O-Tech MG205XPB17) attached to a pressure vessel. Gas and the solution are fed through the pump into the pressure vessel to generate nanobubbles. The pressure in the vessel is controlled by adjusting in and out fluxes of the solution.

**Figure 5:**
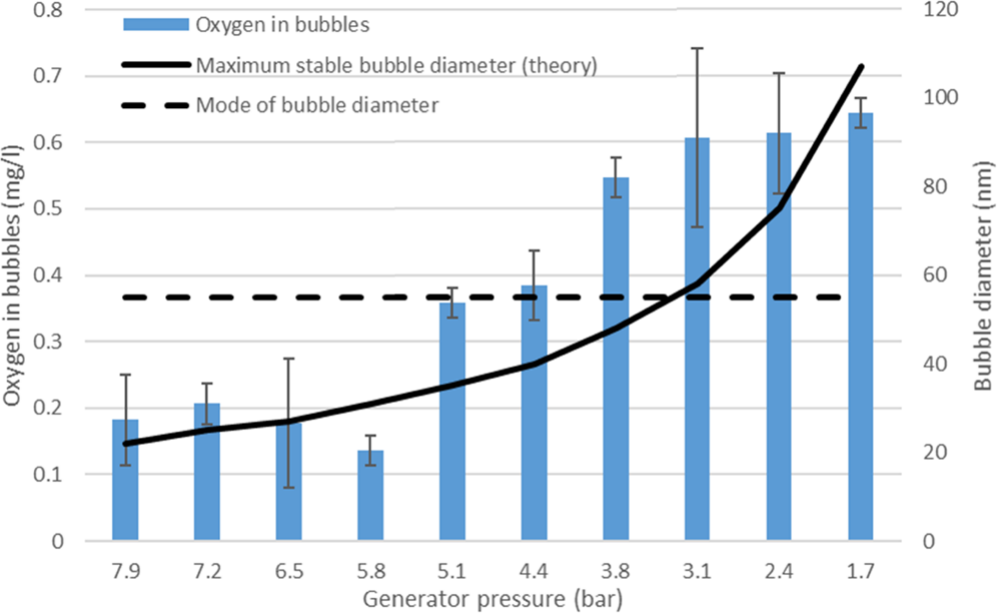
Oxygen content in bubbles with various generator pressures, the maximum calculated metastable bubble diameter by the theory, and the mode of the generated bubble diameter distribution. Error bars represent the standard deviation of three parallel measurements.

Nanobubbles were cured at various pressures. The amount of generated nanobubbles was estimated from the oxygen content of the samples by measuring oxygen content by the Winkler method.^45^ Prior to the nanobubble generation, air was bubbled through the solution to saturate the solution with gas. After bubbling, the solution was treated once with the nanobubble generator. The solution was further treated by cycling 500 mL of the solution through the nanobubble generator for 10 min to enrich nanobubbles in the solution. Oxygen content of the single processed solution was subtracted from the enriched nanobubble solution. The results of the experiments are shown in Figure 5.

According to Figure 5, the amount of gas was relatively constant, between 1.7 and 3.1 bar curing pressures. At higher pressures, the amount of gas decreased. According to the proposed theory, nanobubbles having a diameter 55 nm are unstable over 3.1 bar pressures, which agrees with the observed gas contents. It should be noticed that if the amount of analyzed gas would simply result from gas supersaturation, the amount should increase with higher pressures and the observed effect would be reversed.

## Abbreviations Used

*A*: surface area based on the bubble radius of curvature [m^2^]
*A*_base_: surface area under the spherical cap [m^2^]
*c*: gas concentration in the solution [mol/L]
*A*_cap_: surface area of the cap [m^3^]
*c*_0_: gas saturation concentration [mol/L]
*G*_tot_: Gibbs energy of the studied system [J]
*G*_sol_: Gibbs energy related to dissolved gas [J]
*G*_int_: Gibbs energy related to the gas–liquid interface [J]
*G*_liq_: Gibbs energy related to liquid [J]
*G*_tot_: Gibbs energy related to vapor pressure [J]
*G*_0_: standard Gibbs energy [J]
*h*_sol_: depth of solution [m]
*k*: Henry’s constant [L Pa/mol]
*n*: quantity [mol]
*p*: pressure in a bubble [Pa]
*p*_atm_: atmospheric pressure [Pa]
*p*_0_: external pressure [Pa]
*r*: radius of a bubble [m]
*p*_vap_^∞^: vapor pressure over a flat surface [Pa]
*p*_vap_: vapor pressure over a curved surface [Pa]
r_e_: radius of a bubble in chemical equilibrium [m]
*R*: gas constant [J/(mol K)]
*S*: entropy [J/K]
*T*: temperature [K]
*U*: internal energy [J]
*U*_gas_: internal energy related to the internal gaseous phase [J]
*V*_m_: molar volume [m^3^/mol]
*V*: volume based on the bubble radius of curvature [m^3^]
*V*_total_: Volume of the total system [m^3^]
*V*_liq_: volume of the liquid phase [m^3^]
*V*_cap_: volume of the spherical cap [m^3^]
[deg]; θ: bubble contact angle (measured through the gas) [deg]
θ_*c*_: contact angle [deg]
σ: liquid/gas surface tension [N/m]
σ_s/g_: solid/gas surface energy [N/m]
σ_s/l_: solid/liquid surface energy [N/m]
ρ: density [kg/m^3^]
