# Correction to “Metastable Nanobubbles”

**DOI:** 10.1021/acsomega.1c04288

**Published:** 2021-08-20

**Authors:** Tapio Vehmas, Lasse Makkonen

We wish to correct the following errors in the presentation of equations in our paper.

Equation 10 gives the condition where a bubble remains in the metastable state. However, in the original article, the text above the equation implies the opposite condition, where a bubble dissolves. Therefore, the text above eq 10 should read “When a decrease in the bubble radius causes a negative change in the total Gibbs energy, the bubble will dissolve spontaneously. When the change is positive, the bubble is in the metastable state (eq 10).”

A minus sign is missing from eqs 13 and 14 of the original paper but is correctly included when eq 14 is used in eq 22. In this connection, we wish to clarify that eq 13 describes the energy required to dissolve the gas in the bubble. Accordingly, eq 13 provides the *change* in the Gibbs energy of the gas in the *bubble*, and the term *G*^0^ should not appear in eq 13. Thus, the correct form of eq 13 is
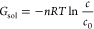
13

Consequently, eq 14 should appear
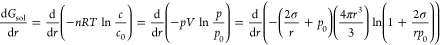
14

From the numerator of the three first terms in eq 20 of the original paper, the differentiation symbol is missing. The correct form is
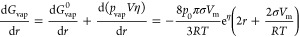
20

In eq 22 of the original paper, a factor 4π/3 is missing from the last term, obtained from eq 14.

With these corrections the final criterion of metastability of a bubble, eq 22, reads
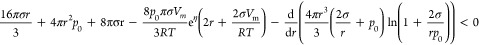
22

The correct form of eq 22 above was the equation used in calculating the results. Therefore, the errors in the formal presentation did not have any effect on the results or the conclusions of the original article. We apologize the readers for the inconvenience caused by these errors in our presentation.

